# Supplementary material for: Research on performance and dynamic competency evaluation of bid evaluation experts based on weight interval number
Source: PLoS One. 2022 Jul 1;17(7):e0269467. doi: 10.1371/journal.pone.0269467 (PMC9249197; doi:10.1371/journal.pone.0269467)
Supplement: Attachment — Submitted filename: Response to Reviewers.docx [file pone.0269467.s001.docx]

Respected PLOS ONE Editors and Reviewers:

Thank you very much for the comments and suggestions for this paper. We have taken these comments and suggestions seriously and modified the paper. Now for your comments and suggestions to respond. Our manuscript number is: PONE-D-21 - 28448 : Research on performance evaluation of bid evaluation experts based on weight interval number theory

**Miguel Angel Sanchez Granero**

**Academic Editor**

**PLOS ONE**

**Journal Requirements:**

**1. Please ensure that your manuscript meets PLOS ONE's style requirements, including those for file naming. The PLOS ONE style templates can be found at**

**https:/journals.plos.org/plosone/s/file?id=wjvg/PLOSOne_formatting_sample_main_body.pdf and**

**https://journals.plos.org/plosone/s/file?id=ba62/PLOSOne_formatting_sample_title_authors_affiliations.pdf**

We have modified the manuscript in the light of PLOS ONE style requirements.

**2.We note that the grant information you provided in the 'Funding Information' and 'Financial Disclosure' sections do not match.** **When you resubmit, please ensure that you provide the correct grant numbers for the awards you received for your study in the 'Funding Information' section.**

We reconfirmed the funding information.

**3. We note that you have stated that you will provide repository information for your data at acceptance. Should your manuscript be accepted for publication, we will hold it until you provide the relevant accession numbers or DOIs necessary to access your data. If you wish to make changes to your** **Data Availability statement, please describe these changes in your** **cover letter and we will update your Data Availability statement to reflect the information you provide.**

We have modified the data availability statement in the cover letter.

**4.PLOS requires an ORCID iD for the corresponding author in Editorial Manager on papers submitted after December 6th, 2016. Please ensure that you have an ORCID iD and that it is validated in Editorial Manager. To do this, go to 'Update my Information' (in the upper left-hand corner of the main menu), and click on the Fetch/Validate link next to the ORCID field. This will take you to the ORCID site and allow you to create a new iD or authenticate a pre-existing iD in Editorial Manager, Please see the following video for instructions on linking an ORCID iD to your Editorial Manager account: https://www.youtube.com/watch?v=_xcclfuvtxQ**

The corresponding authors have applied for ORCID iD and verified.

**5.We note you have included a table to which you do not refer in the text of your manuscript. Please ensure that you refer to Table 3 & 6 in your text; if accepted, production will need this reference to link the reader to the Table.**

Tables 3 and 6 in this article are not referenced, It is the author’s error and have been modified.

**Review Comments to the Author**

Reviewer #1:

This article presents a performance evaluation index system for bid evaluation experts and a corresponding evaluation model based on the calculation of the number of index weight intervals, However, I think the quality of the paper is not enough to meet the academic requirements, and there is still a lot of room for improvement. My comments to you generally represent a class of problems that the author needs to make serious revisions to the full text. Representative questions are as follows:

**1.The novelty of the work is not very clear. The introduction does not reflect the importance of the research problem.**

We have modified the introduction part of the manuscript, and mentioned the main problems that currently exist : ①Bid evaluation experts into the pool after the cycle of assessment issues, ②the limitations in performance management of bid evaluation experts after participating in project evaluation.

**2.The literature review of the paper lacks systematicness and logic, the selection of references is not focused enough, and many papers are not related to the research topic of the paper.**

We have revised the literature review, and also modified the Corresponding references.

**3.The paper can not explain the superiority of the proposed method and can not reflect the innovation of the research work.**

We have revised the literature review and pointed out the shortcomings of the literature we referenced to construct our evaluation model. We prove our point of view in the evaluation model, and justify our choice to use the number of weight intervals, and compare them in the empirical analysis to prove the superiority of our method.

**4. "Offline bidding evaluation and online bidding evaluation are two common bidding evaluation methods"', this statement is not correct!**

We agree with the reviewer ’ s view and have revised it.

**5. Important statements lack literature support.**

We rechecked the text and modified the contents of the text.

**6.There are some grammar errors and statements are still not clarity. e.g. the last sentence in the first section of the introduction.**

We thoroughly re-edited the article to correct our grammatical errors.

**7.There are some format errors in Reference.**

We have modified the references with reference to the style of PLOS ONE.

**8.In the Description section, the writing format is not standardized.**

We have modified the manuscript format with reference to the style of PLOS ONE.

**9.The authenticity and reliability about data in Empirical Analysis need to clarified furtherly.**

The data in the empirical part is obtained through research to simulate the basic data of bid evaluation experts' performance and dynamic competency characteristics, and then simulate the bid evaluation experts who meet the actual situation for analysis, the authenticity and reliability of the data can be guaranteed

**Reviewer #2:**

**1)The Abstract and Introduction sections should be improved. The information given by the manuscript is generally self-contained. However, there should be some improvements regarding its contents with necessary amplifications. There is lack of enough illustrations regarding the necessity of introducing performance evaluation of bid evaluation experts. I would suggest authors to provide a table to analyze the literature review. This will benefit the understanding the research gaps and your plans to fill them, The table will also help in comparing the existing models in the literature and highlighting its contributions in the literature. Please work on improving the clarity of your paper.**

We carefully revised the abstract and introduction sections. We also revised the analysis in the literature review section. The necessity of performance evaluation and dynamic competency evaluation of bid evaluation experts has been highlighted. And we have explained the shortcomings of previous studies and innovation of this article. It is indeed an intuitive and effective method to analyze the literature review through tabular analysis. However, we believe that at present, there are few studies on bid evaluation experts, and the verbal descriptions can already be expressed clearly, so your method is not adopted.

**2)There are many existing publications in this research area. It is not clear the authors collected these papers based on which criteria. The review in the Introduction is too general. The historical review of the bid evaluation is weak, Authors are suggested to read some comprehensive and relevant publications on the existing counterparts to highlight the necessity of using weight interval number theory. For instance, some of them are proportional hesitant fuzzy linguistic term set and HFLTS possibility distribution, etc.**

We have reworked the introduction to enhance the review of bid evaluation from two aspects: the importance of bid evaluation and the important role of bid evaluation experts. The reason why we use the weight interval number is also re-stated in the literature review: namely, we construct a mathematical optimization model conditional on the weight interval number according to clustering distinction in competency evaluation with reference to previous ideas.

**3) The level of English about this manuscript does not meet the journal's desired standard., Therefore, language should be greatly improved, There are too many grammatical mistakes and typos, Please carefully revise and improve it. The paper requires a thorough editing.**

We are sorry that our writing style has troubled you. We have re-edited the paper thoroughly to correct our grammatical mistakes and spelling errors.

**4)The author should state the source of the data and whether it is realistic data and how the expression of the presented measures for uncertainty being evaluated is useful for solving current real-life problems. More elaborations on these aspects are suggested.**

We are sorry that our writing errors has caused you trouble. We have re-edited the paper thoroughly to improve our grammatical errors and spelling mistakes.

**5) The comparison analysis and in-depth discussions seem to be casual in this paper. Please enhance them to demonstrate the reliability of your advocated model. The related and recent work should be discussed and commented on. Some of them are: Bid evaluation in civil construction under uncertainty: A two-stage LSP-ELECTRE III-based approach and Bid Evaluation for Major Construction Projects Under Large-Scale Group Decision-Making Environment and Characterized Expertise Levels.**

It is possible that our lack of clarity or our errors in grammar and spelling led to your misunderstanding. We have re-edited it and also explained it in the text. However, we still need to explain your question here: we did cite the ideas in “Bid evaluation in civil construction under uncertainty: a two stage LSP-ELECTRE III-based approach” At the same time, you also mentioned another paper, “bid evaluation for major construction projects under large scale group decision making environment and characterized expertise levels”, However, our research perspective is different from these two papers. These two papers mainly focus on the classification, consensus and collective decision-making of experts in the process of bid evaluation. However, our research angle is different from these two papers, which are mainly about the issues of expert classification, consensus building and collective decision making in the bid evaluation process, but we evaluate the bid evaluation experts by constructing the performance and dynamic competency evaluation index system and evaluation model, and the experts in the process of judging the importance of the evaluation index system are composed of relevant stakeholders, not exactly bid evaluation experts. . The main reference document for constructing the evaluation model is “Impact of discrimination performance of aircraft attitude indicator on aviation safety”. We have improved the shortcomings of this article, and also argued the advancement and reliability of this paper in detail in the comparative analysis section.

**6)The managerial implications of this research should be enhanced. How decision or policymakers can benefit from this work with robust and reliable conclusions. What will change the main insights if different methods were introduced.**

We have added the Management Enlightenment section to clarify how relevant management can benefit from this work.

**7) The conclusion should be improved to summarize clearly the main contributions of the paper and future research efforts. It will increase the impact of the paper if the authors try to indicate this explicitly in the manuscript. Critical limitations in the proposed framework should be offered. Extensions and applications of the proposal in other fields could be exemplified in the Conclusion section.**

We revised the conclusions, summarized the main contributions and limitations of the paper, as well as future research work.

Thanks again to the academic editors and two reviewers for their comments and suggestions on my manuscript, which is of great help to improve the quality of my article.
