# Supplementary material for: Research on performance and dynamic competency evaluation of bid evaluation experts based on weight interval number
Source: PLoS One. 2022 Jul 1;17(7):e0269467. doi: 10.1371/journal.pone.0269467 (PMC9249197; doi:10.1371/journal.pone.0269467)
Supplement: Attachment — Submitted filename: Response to Reviewers.docx [file pone.0269467.s002.docx]

Dear Editors and Reviewers:

Thanks for your reply, we have revised the paper again. Now I will respond to your suggestions and comments. Our manuscript number is: PONE-D-21-28448R1: Research on performance and dynamic competency evaluation of bid evaluation experts based on weight interval number

**Miguel Angel Sanchez Granero**

**Academic Editor**

**PLOS ONE**

**Journal Requirements:**

**1.Please review your reference list to ensure that it is complete and correct. If you have cited papers that have been retracted, please include the rationale for doing so in the manuscript text, or remove these references and replace them with relevant current references. Any changes to the reference list should be mentioned in the rebuttal letter that accompanies your revised manuscript. If you need to cite a retracted article, indicate the article’s retracted status in the References list and also include a citation and full reference for the retraction notice.**

The papers we cite do not contain papers that have been retracted; the cited papers can be found in the relevant databases. We have modified the format of the cited papers by referring to the style of PLOS ONE, but some of them do not have DOI or PMID, and we have provided accessible links.

**Comments to the Author**

**3. Has the statistical analysis been performed appropriately and rigorously?
Reviewer #1: Yes**

**Reviewer #2: N/A**

Because of the confidentiality of bid evaluation experts, we have no way to obtain the real data, and our paper only roughly understands the performance and dynamic competence characteristics of bid evaluation experts, so that the virtual bid evaluation experts can basically match the actual situation, and therefore we did not conduct a rigorous statistical analysis

**Review Comments to the Author**

Reviewer #1:

**Compared with the previous version, the paper has been improved greatly, but there is still much room for improvement in some areas. The literature review of the paper is not sufficiently relevant and focused compared with the core issues of the paper. Management inspiration and conclusion recommendations are put together.**

Thank you for acknowledging our first revision, and this time we have carefully considered your suggestions, which we believe to be sound. Therefore, we have revised the literature review section to highlight our research questions, and we have also put together management inspiration and concluding recommendations.

Reviewer #2:

**The authors respond well to my comments, I think it is greatly improved and is ready for publication in the current form.**

Thank you for your approval of our paper.

Thanks again to the academic editors and two reviewers for their comments and suggestions on my manuscript, which is of great help to improve the quality of my paper.
